# A Decision Aid to Support Vocational Rehabilitation Professionals Offering Tailored Care to Benefit Recipients with a Long-Term Work Disability: A Feasibility Study

**DOI:** 10.1007/s10926-023-10105-7

**Published:** 2023-04-10

**Authors:** Christa J. C. de Geus, Maaike A. Huysmans, H. Jolanda van Rijssen, Trees T. Juurlink, Marianne de Maaker-Berkhof, Johannes R. Anema

**Affiliations:** 1 Department of Public and Occupational Health, Amsterdam Public Health Research Institute, Amsterdam UMC, Vrije Universiteit Amsterdam, Van der Boechorststraat 7, NL, 1081 BT Amsterdam, The Netherlands; 2 Dutch Institute of Employee Benefit Schemes (UWV), Amsterdam, The Netherlands; 3 Research Centre for Insurance Medicine, AMC-UMCG-VUmc-UWV, Amsterdam, The Netherlands

**Keywords:** Return to work, Long-term sick leave, Vocational rehabilitation, Decision aid, Work disability pension

## Abstract

**Purpose:**

This feasibility study focusses on the implementation and use of a decision aid, which supports vocational rehabilitation (VR) professionals in helping clients with a disability pension return to work in practice. The decision aid shows an overview of the clients’ return to work barriers and suggests suitable VR interventions based on these barriers.

**Methods:**

The study population consisted of VR professionals working at the Dutch Social Security Institute and their clients receiving a (partial) work disability pension. The feasibility was measured with concepts of the Linnan and Steckler framework and the attitude, social norm and self-efficacy model. Data were collected using questionnaires, checklists and qualitative interviews.

**Results:**

Ten professionals participated in this study. Fifty-four clients were asked to fill in the questionnaire of the decision aid and 32 clients received VR care based on the decision aid. In general, VR professionals and clients were satisfied with the decision aid and perceived a few barriers for using the decision aid.

**Conclusions:**

This study showed that it is feasible to implement and use the decision aid. To improve the implementation of this decision aid, it should be implemented in digital systems used by professionals to improve efficiency of working with the decision aid.

**Supplementary Information:**

The online version contains supplementary material available at 10.1007/s10926-023-10105-7.

## Introduction

Returning to work not only limits the loss of income, but being employed is also beneficial for (mental) health [1–3], quality of life [4], and well-being [5]. Therefore, in many countries efforts are made to support people in returning to work. Many people receiving a partial work disability pension do not return to work [6–8]. However, returning to work with a disability is far from easy, due to personal health, or external circumstances. To help people (partially) return to work, it is therefore important that they receive effective vocational rehabilitation (VR) interventions tailored to their situation. VR professionals however have limited time and resources for this [10].

To improve evidence-based working of VR professionals, several tools have been developed (e.g. [11–13]). Despite these efforts, there is still a lack of standardized procedures, guidelines or tools to support VR professionals in identifying relevant RTW barriers and choosing effective VR interventions for work disability pension recipients. To support the quality of the assessment of important RTW barriers and to improve standardized procedures, we have developed a decision aid [9] based on the ICF-model [14]. This decision aid supports in assessing RTW barriers and facilitators based on the clients’ answers on a questionnaire, and in offering tailored VR care by suggesting relevant evidence-based VR interventions. Our earlier study showed that this decision aid was eff ective in increasing agreement with a gold standard (supplementary material 1). This way, the decision aid improves evidence-based practice among VR professionals.

The present study describes the feasibility of using our decision aid by VR professionals in practice. Our main research aims were 1) to investigate to which extent the protocol steps of using the decision aid in practice were realized by VR professionals and their work disability pension recipients (clients); 2) to assess the barriers and facilitators for using the decision aid in practice; and 3) to assess the attitude of VR professionals and clients towards the decision aid.

**Table:** 

Text block 1 In the Netherlands, workers on sick leave for at least two years can apply for a work disability benefit at the Dutch SSI. After two years, employers can terminate the contract of these people. An insurance physician conducts a medical disability assessment to evaluate if a work disability benefit is applicable. A labour expert then calculates the percentage of work disability (WIA WGA). If people have remaining work capacity or if an improvement in work capacity can be expected in the future, people receive help from the SSI with (partially) returning to work. A VR professional assesses the situation of the client and suggests one or multiple suitable VR interventions. The VR intervention is carried out by a third party. Progress is supervised by the VR professional of the Dutch SSI.	

## Methods

### Study Design

A feasibility study was performed between December 2021 and May 2022. Components of the Linnan & Steckler [15] framework and determinants of behavior by using the attitude, social norm and self-efficacy model (ASE model), derived from the Theory of Planned Behaviour [16] were collected using semi-structured interviews, checklists and questionnaires. VR professionals of the Dutch Social Security Institute (SSI) were recruited to participate in this study**.** Both occupational rehabilitation officers and labour experts could participate. Types of education of the VR professionals vary. Compared to occupational rehabilitation officers, labour experts have an additional one-year specialization as a labour expert***.*** Participating professionals were asked to use the decision aid in the VR assessment of circa five clients receiving a work disability pension. The VR professionals were trained (June–September 2021) in a previous experimental study (supplementary material 1) in the use of the decision aid. Before the start of the feasibility study, VR professionals were visited by the researchers (November–December 2021) to further train them in using the decision aid in practice. Data were collected on several moments (shown in text block 2) from both professionals and clients, using questionnaires, a checklist and interviews. An overview of the interview guides can be found in Appendix 1.

**Table:** 

Text block 2: Data collection (1) At the start of the training session VR professionals filled in a questionnaire to retrieve their characteristics (2) Immediately after the training session, VR professionals filled in a questionnaire to evaluate the training session. (3) After each client that was asked to fill in the questionnaire, the VR professionals filled in a checklist to measure reach and dosage. (4) After including their last client, VR professionals filled in an evaluative questionnaire to measure the level of satisfaction, barriers and facilitators for using the decision aid, attitude, self-efficacy, intention for future use, and knowledge and skills with regard to the decision aid. (5) After clients had received a VR assessment with the decision aid, VR professionals asked clients to participate in an interview with the researchers about their experiences. and satisfaction with filling in the questionnaire for the decision aid and with the VR assessment they received. (6) After VR professionals included their last client and completed the evaluative questionnaire, VR professionals were asked to participate in an interview to gain in-depth knowledge on their level of satisfaction and experience, attitude, intention for future use, knowledge and skills, self-efficacy and potential barriers and facilitators.	

This study was approved by the Medical Ethics Committee of Amsterdam UMC, VU University Medical Centre Amsterdam (2021.0406). The committee declared that no comprehensive ethical approval was needed. All participants signed informed consent before participation.

### Study Population

#### VR Professionals

VR professionals were eligible for this study if they (1) were employed at the Dutch SSI, (2) had at least 6 months experience with the vocational rehabilitation of clients receiving a work disability pension, (3) conducted VR assessments with these clients, and (4) participated in the experimental study. in which they were trained to use the decision aid.

#### Clients

Clients were eligible to participate in this study if they received a work disability pension (WIA WGA) and were appointed to a VR professional that participated in the study. Clients were recruited for the interviews with help of the VR professionals. VR professionals informed clients about the opportunity to participate in an interview on a voluntary basis. To indicate their willingness to participate in the interview, clients could fill in the informed consent and send it to the researchers, or give consent to the VR professional to distribute their name and telephone number to the researchers. If more clients than needed indicated they wanted to participate in the interview, purposive sampling was used to select clients spread across the participating VR professionals. We included clients for interviews until data saturation was reached [17].

### Intervention: Decision Aid

The decision aid was based on the ICF-model [14] and the results of a previous Delphi Study [9]. The aim of the decision aid was to support VR professionals in delivering tailored care to improve return to work of work disability pension recipients with remaining work capacity. The decision aid supports in identifying potential RTW barriers of the client and suggests relevant directions for VR interventions to target these barriers. The VR trajectories offered at the Dutch SSI and the VR intervention directions in the decision aid differ, as described in text block 3.

**Table:** 

Text block 3
At the Dutch SSI the VR interventions are grouped together in overarching VR trajectories. The intervention directions in the decision aid are not fully linked to these overarching trajectories because available interventions differ per SSI office. Therefore, the VR directions suggested by the decision aid are aimed at targeting a certain barrier and are more generally formulated than the VR trajectories used in practice. In this study, VR professionals can choose one of the overarching VR trajectories of the SSI themselves and use the decision aid to describe which specific VR directions for interventions, based on availability within the district, should be offered to the client.

The decision aid consists of five protocol steps, which are presented in Table 1. For this study, VR professionals sent the questionnaire to the client either by post or by using the SSI’s digital environment. After the questionnaire was filled in and returned, the VR professionals had to fill in the answers of the client in the decision aid. For feasibility of this study, the decision aid was built in Microsoft Excel.

**Table 1:** Protocol steps of using the decision aid by professional

1. Questionnaire
Ask the client by telephone to fill in questionnaire
Send questionnaire and information to the client with the invitation for the VR assessment
Receive questionnaire that is returned filled in by the client
Receive questionnaire that is filled in correctly by the client (e.g. not giving multiple answers on a question and readable answers according to the VR professional)
2. Preparation of VR assessment by VR professional
Analyze the answers of the client on the questionnaire and the results of the decision aid on which factors are barriers
3. VR assessment with client: prioritizing barriers
Discuss the answers of the client on the questionnaire and the results of the decision aid (which factors are barriers and which factors are facilitators) with the client
Select most important RTW barriers (max. 3) in consultation with the client
Select RTW barriers that are suggested by the decision aid or give arguments for deviating from the decision aid
4. VR assessment with client: choosing suitable VR intervention(s)
Discuss VR interventions that are suggested by the decision aid with the client
Select the most suitable VR interventions (max. 3) in consultation with the client
Select VR interventions that were suggested by the decision aid or give arguments for deviating from the decision aid
5. Documentation
Document outcomes of decision aid

### Training Session VR Professionals

VR professionals were trained in the use of the decision aid in one day. During the training session, they received instructions on how the decision aid was developed and what the protocol steps (Table 1) were. After this, VR professionals practiced working with the decision aid using several case vignettes. An overview of the learning objectives can be found in Table A1 in Appendix 2.

### Data Collection

Data on the components of the Linnan and Steckler framework [15] and the ASE-model [16] were collected using quantitative and qualitative methods (i.e. checklists, questionnaires and interviews). An overview of the definition, operationalization and data collection of the components can be found in Table 2.

**Table 2:** Process measures and data collection

Key component	Definition	Operationalization	Data collection
Recruitment	*Professionals and clients* Procedures and number of initially recruited VR professionals and clients Representativeness and characteristics of the VR professionals and clients Reasons for non-participation	N/A	Researcher logs and checklist*
Reach	*Professionals* Number of professionals that participated in the study as proportion of number of professionals that were invited to participate *Clients* Number of clients that filled in the decision aid as proportion of clients invited to fill in the decision aid	N/A	Researcher logs and checklist*
Dosage	*Professionals* Extent to which the protocol steps were completed	A learning goal was adequately met if more than 75% of the participants scored (4) agree or (5) totally agree For each client, the VR professional indicated which protocol steps (Table 1) were completed. Per protocol step the total number of clients for which the step was completed was calculated Only clients that filled in and returned the questionnaire, were included in these analyses	Checklist* and semi-structured interviews** with VR professionals
Satisfaction and experience	*Professionals* Satisfaction and experience with the decision aid itself, with the work process of the decision aid and with the influence of the decision aid on their work compared to the usual situation *Clients* Satisfaction and experience with the questionnaire, with the decision aid and with the VR assessment	∙ “On a scale of 1 to 10, which grade would you give the decision aid” ∙ “On a scale of 1 to 10, which grade would you give the work process based on the decision aid?” ∙7 items on the extent to which the decision aid improved the different steps of the current work process. Answers were given on a five-point Likert-scale ranging from ‘Fully disagree’ to ‘Fully agree’	Evaluative questionnaire and semi-structured interviews** with VR professionals and clients
Barriers and facilitators	*Professionals and clients* Barriers and facilitators for the use of the decision aid	13 items of the ‘barriers and facilitators assessment instrument’ (Peters et al. 2002) An example item is: “ This decision aid gives me enough space to make my own assessments” Answers were given on a five-point scale ranging from ‘Fully disagree’ to ‘Fully agree’ Items 1, 11,12 and 13 were reverse-coded	Evaluative questionnaire and semi-structured interviews** with VR professionals and clients
Attitude	*Professionals* Attitude towards the use of the decision aid in daily practice	9 items based on a scale developed by Zwerver et al., 2013 [25] An example item is: “This decision aid supports in making complex decisions” Answers were given on a five-point scale (Fully disagree-Fully agree)	Evaluative questionnaire and semi-structured interviews** with VR professionals
Intention for future use	*Professionals* Intention to use the decision aid in daily practice	7 items based on a scale developed by Zwerver et al., 2013 [25] An example item is: “I have the intention to use the decision aid or parts of the decision aid in the future” Answers were given on a five-point scale (Fully disagree-Fully agree)	Evaluative questionnaire and semi-structured interviews** with VR professionals
Self-efficacy	*Professionals* Self-efficacy in the use of the decision aid in daily practice	10 items based on a scale developed by Zwerver et al., 2013 [25] An example item is: “I feel I have enough skills to use the decision aid in practice” Answers were given on a five-point scale (Fully disagree-Fully agree)	Evaluative questionnaire and semi-structured interviews** with VR professionals
Knowledge and skills	*Professionals* Self-perceived knowledge and skills to use the decision aid in daily practice	6 items based on a scale developed by Zwerver et al., 2013 [25] An example item is: “I have enough knowledge to use the decision aid in practice” Answers were given on a five-point scale (Fully disagree-Fully agree)	Evaluative questionnaire and semi-structured interviews** with VR professionals

*A checklist was used to measure recruitment, reach and dosage

**An overview of the interview guide is given in Appendix 1

### Data Analysis

The quantitative data were analyzed using descriptive statistics. The qualitative interviews were conducted by author CdG and author MdMB using a deductive approach. The interviews were recorded and then summarized by MdMB and verified by CdG. A coding scheme was constructed to categorize the information of the summarized interviews. The quantitative results were connected to the qualitative results using manifest content analysis. The unit of analysis was sentences or short paragraphs with sentences describing the same topic. CdG conducted the first round of coding and discussed the coding with MDMB. After this, the codes were categorized according to the components in this study (Table 2). The categorization was discussed until consensus was reached with MDMB. The results were then discussed with all authors. The results of the qualitative analysis were used to support the quantitative results. Per theme, relevant quotes were retrieved from the interviews.

## Results

### Recruitment and Reach

#### VR Professionals

In June 2021, twelve of the approached 24 VR professionals agreed to participate. Reasons for non-participation were: not meeting the inclusion criteria (e.g. currently not conducting VR assessments with clients with a work disability pension), and not enough time to participate in the study. Four participants of the twelve participating professionals dropped out of the study before including clients due to sickness, new work role, not having assessments with eligible clients, or no time to participate in the study.. To reach the planned number of ten participants, two additional VR professionals were recruited and trained separately. They did not evaluate the training, because they received a shorter version of the training (without case vignettes). In total ten VR professionals participated in this feasibility study and five of them were interviewed. See Table 3 for their characteristics.

**Table 3:** Characteristics of VR professionals and clients

	VR professionals		Clients	
	N	Median (Range)	N	Median (Range)
Gender				
Male	1		2	
Female	9		6	
Age		52 (39–63)		47,5 (39–60)
Years of working experience with long-term disabled workers		6 (2–20)		

#### Clients

In total, ten VR professionals asked 54 clients to fill in the questionnaire of which 36 (67%) reacted. Reasons for non-response were: 1) Based on the telephone call the VR professional decided not to send the questionnaire, because the client indicated that he/she did not want to fill in the questionnaire, or because the VR professional thought the client was too vulnerable. 2) The questionnaire was not received on time by the client or returned on time to the professional. 3) The client received the questionnaire, but did not fill in the questionnaire due to personal circumstances such as: physically not able, mental problems, or thinking the questions were to personal. Thirty-two of the 36 clients received a VR assessment based on the decision aid. For four clients this was not the case, because the client still received treatment, the VR professional did not have enough time to discuss the questionnaire, or the VR professional did not receive the questionnaire on time. As a result, 32 of the 36 clients that filled in the questionnaire, received a VR assessment based on the decision aid.

Seventeen clients (53%) of the 32 clients for which the decision aid was used, agreed to be interviewed, of whom eight clients were selected by the researchers. See Table 3 for their characteristics.

### Training Session

All eight participants that evaluated the training rated the training with a 7 or higher (on a scale of 1–10, median = 8, range = 7–9). VR professionals especially appreciated the structure and the clarity of the training session. However, they also mentioned that time was too limited to properly practice with all the case vignettes and suggested to include more participants in the training session (training sessions included two to four VR professionals). Participants agreed that all learning objectives (Appendix 2) were met (range 4.0–4.4 on a scale of 1–5).

### Dosage

Table 4 provides an overview of the number of clients and the extent to which protocol steps were completed.

**Table 4:** Dosage of decision aid

Protocol step	Yes	No	Reasons for not completing protocol step
Client filled in questionnaire of the decision aid (in time) (N = 36)			
1. Questionnaire			
Client was asked by telephone to fill in questionnaire, before questionnaire was sent	33	3	No specific reasons mentioned
Questionnaire and information was sent to client with the invitation for the VR assessment	35	1	Client was not ready to receive a VR assessment at the first meeting. Client received the questionnaire in the second meeting 5 weeks later
Client returned a complete questionnaire	34	2	One client left one question unanswered. Another client left four questions unanswered
Client filled in questionnaire correctly (e.g. not giving multiple answers on a question and readable answers according to the VR professional)	30	6	Clients that did not fill in the questionnaire correctly, made a mistake (e.g. giving multiple answers on a question) in two to six questions
VR professional used decision aid in VR assessment (N = 32)			
2. Preparation of VR assessment			
Professional analyzed the answers of the client on the questionnaire and the results of the decision aid on which factors are barriers	27	5	VR professional had too little time to prepare the VR assessment, due to not receiving the questionnaire on time or preference to hear the story of the client him- or herself to earn the trust of the client
3. VR assessment with client: prioritizing barriers			
Professional discussed the answers of the client on the questionnaire and the results of the decision aid (which factors are barriers and which factors are facilitators) with the client	26	6	Personal circumstances of the client or not having received the questionnaire on time
Professional selected most important RTW barriers in consultation with client	21	11	According to the VR professional, clients were not ready to receive a VR intervention, due to personal circumstances of the client or because the client already had a job
Professional selected RTW barriers that were also suggested by the decision aid	29	3	VR professionals selected other RTW barriers than suggested, because of barriers that became clear during the meeting or because of circumstances of the client
4. VR assessment with client: choosing suitable VR intervention(s)			
Professional discussed VR interventions suggested by the decision aid with client	25	7	VR professionals thought the client was not eligible to receive a VR intervention. Several arguments were given: client has already received a VR intervention, client is at the moment not ready to receive a VR intervention. VR professionals often did plan a new meeting to discuss a relevant VR intervention
Professional used decision aid to choose the most suitable VR interventions in consultation with client	21	11	VR professionals did not have enough time during the interview to discuss VR interventions, the personal situation of the client or specific wishes of the client
Professional selected VR interventions that were also suggested by the decision aid	17	15	No additional specific reasons mentioned
5. Documentation			
Outcomes of decision aid were documented	23	9	For some clients, the VR professionals did not do this, because the Excel file with the decision aid was too slow due to technical difficulties

#### Questionnaire of the Decision Aid

Thirty-six clients filled in the questionnaire of the decision aid. In most cases, clients were (n = 33) informed by telephone about the questionnaire. Some VR professionals perceived these calls as time-consuming, because clients started to explain their personal situation during the phone call. Most clients returned a complete questionnaire (n = 34) and filled in the questionnaire correctly (n = 30). When information was missing, VR professionals discussed this with the clients during the VR assessment and added the information to the decision aid.

#### Preparation of VR Assessment

The VR professionals did not always prepare for the VR assessment with the use of the questionnaire and the results of the decision aid. Reasons were that the VR professional had too little time to prepare the VR assessment because he did not receive the questionnaire on time, or a preference to hear the story of the client himself.

#### VR Assessment with Client: Prioritizing Barriers

With 26 of 32 clients, VR professionals first discussed the results of the decision aid (which factors are barriers for RTW), according to the protocol. VR professionals mentioned that clients often recognized themselves in the outcomes of the decision aid. VR professionals had different strategies of discussing the RTW barriers. Some VR professionals discussed all factors (including the facilitators), others only discussed the RTW barriers. As one VR professional explains:

I did not discuss all questions, only things that stood out to me and of which I thought: hmm…; then I asked. For example, one person left something unanswered [in the questionnaire]. Well, then [after asking] information came up that I did not know from the file. So I could discuss this more in depth. So…, someone happened to do voluntary work, and I found it strange that this had never came up during a previous assessment, but it did in to the questionnaire. And that is very valuable, right? I always think that this [voluntary work] will come up during the assessment, because someone doing voluntary work is in a different position than someone not doing voluntary work. (VR professional 2)

For 21 clients the most important barriers were selected in consultation with the client. Some VR professionals selected the most important RTW barriers and then discussed these with the client to see if the client agreed these RTW barriers were indeed the most important. If this was not the case, the VR professional changed the most important RTW barriers in the decision aid. Other professionals first discussed the barriers and then, in discussion with the client, choose the most important RTW barriers:

Especially the questions that were highlighted in red, that is what you pay attention to, right? Sometimes it turned out not to be important for the client to act upon it [the red highlighted barrier] and sometimes it was. So in general I decided together with the client what was the most important to start working on. But that was guided by the answers, well, by the questionnaire actually. (VR professional 1)

Some VR professionals did not select barriers due to (1) the VR professionals’ conviction that the client was not ready, (2) not having enough time during the first meeting to discuss the barriers, (3) the personal situation of the client, or (4) specific wishes of the client.

#### VR Assessment with Client: Choosing Suitable VR Interventions

In most of the cases the VR professional discussed the VR interventions that were suggested by the decision aid (n = 25). If the client was deemed not ready to receive a VR intervention, VR professionals often planned new meetings to discuss suitable VR interventions.

For two thirds of the clients (n = 21) VR professionals chose one or more suitable VR interventions. VR professionals perceived it as more difficult to use the decision aid for this step, than for determining the most important RTW barriers. The main reasons were that the VR interventions were formulated differently from usual care of the SSI, and the complexity of clients’ problems. Some VR professionals used the decision aid to check their own ideas, others followed the decision aid to decide upon suitable VR interventions.

For more than half of the clients (n = 17) VR professionals selected VR interventions that were also suggested by the decision aid. Sometimes, VR professionals deviated from the decision aid.

#### Documentation

For 23 clients the outcomes of the decision aid were documented. VR professionals used the decision aid to explain their choice for a VR intervention:

Yes, and just like I said, I used it for the method to work in an evidence based way. Yes, so actually also just, (…) to explain why I did what I did. (VR professional 3)

#### Satisfaction and Experience

##### VR Professionals

After using the decision aid in practice, all VR professionals rated the decision aid overall with a 7 or higher (range 7–8, median 8, on a scale of 1–10). Working with the decision aid in contact with the clients was rated with a 7 or higher by eight VR professionals, the two other VR professionals rated this with a 5 or a 6 (range 5–9, median 7.5, on a scale of 1–10). According to the VR professionals, the advantages of using the decision aid during the VR assessment outweighed the extra time it took. All VR professionals appreciated the complete overview the decision aid provided of the client before the first meeting. VR professionals noted that with the support of the decision aid, they could better identify the problems of the client (n = 10 (fully) agreed), conduct the VR assessment (n = 7 (fully) agreed) and use the perspective of the client in the assessment (n = 7 (fully) agreed), compared to usual care. A VR professional described the advantages of the questionnaire as follows:

It gives a good insight in the opinion of the client on vocational rehabilitation. I can focus my conversation on this, which is more efficient. And, on top of this, in my experience, the client feels more heard. The client has the feeling that he or she is in control, which gives me the idea that this [decision aid] is of added value, because the client, has a different attitude during the conversation. (VR professional 3)

Using the decision aid did not improve selecting suitable VR interventions for the client compared to usual care (n = 6 neither agreed nor disagreed). VR professionals had mixed opinions on how the VR interventions are presented by the decision aid. Some VR professionals wanted the VR interventions to be more fitting with the overarching VR trajectories currently available at the SSI to offer the client. However, other VR professionals liked the opportunity to use the information to find a fitting VR intervention/ VR trajectory, while still being able to make their own decisions:

It would be easier if the decision aid would tell colleagues: this specific trajectory, or that trajectory. (…) but I actually expect from people who perform these assessments, that they will think for themselves. (VR professional 2).

Considering the VR interventions, it is good that you can check yourself which products we have that we can use. (…) It is better to look more at the VR intervention directions than at products that already exist. (VR professional 4)

VR professionals also indicated that the decision aid did not improve informing the client on VR interventions compared to usual care (n = 4 neither agreed nor disagreed, n = 2 disagreed). An overview of all statements and the responses is presented in Table 5.

**Table 5:** added value of the decision aid compared to the usual situation n = 10

	(Fully) disagreed	Neither agreed nor disagreed	(Fully) agreed
Compared to usual care, using the decision aid improves:			
1. Identifying the problems in different domains of life of the client	0%	0%	100%
2. Conducting the VR assessment	20%	10%	70%
3. Selecting suitable VR interventions	0%	60%	40%
4. Informing the client about the VR interventions	20%	40%	40%
5. Supporting the selection of VR interventions with arguments	10%	30%	60%
6. Including the perspective of the client in the advice	10%	20%	70%
7. Documenting the results of the VR assessment	0%	40%	60%

##### Clients

Clients were satisfied with the decision aid. Most questions were clear to them and not too much of a burden to answer. Some clients regretted that they did not receive a copy of their answers on the questionnaire or an overview of the outcomes of the decision aid. Also, some clients mentioned that they missed the option to explain their answers in the questionnaire:

Filling in the questionnaire went quite smooth, actually. Not everything was equally…, well let me say it like this, …it was clear, but it was not so easy to give a clear answer. You are thinking: on one hand it’s yes, and on the other hands it’s no. Therefore, I made a comment at the end of the questionnaire about some questions of which I thought, well, I could not give a clear answer. At the end, I made a note for her [the VR professional]. (Client 1)

For some people, with medical conditions that influence their energy level, filling in the questionnaire was a burden:

There were many questions (…) but I knew already that it was quite a task. (...) So I discussed with [the VR professional] that I thought it was a lot, but that could also be me, because my energy level is very low. And I also have [physical complaints] so it was quite a burden for me, so to say. (Client 2)

For some clients the questions felt inappropriate for their situation. One client already had a job and another client thought the questionnaire was not appropriate for his level of work capacity.

Clients appreciated that the VR professional already had information about their situation before the start of the meeting:

He [the VR professional] knew of course about my situation and that was pleasant, because otherwise I would get someone on the phone and tell my story again. And now you can take your time and think about it on paper, and then put it away again and [a little later] get back to it. (Client 2)

Clients felt they had the opportunity to explain their answers in the meeting with the VR professional. They also felt heard and felt involved in the process of choosing RTW barriers and a fitting VR intervention.

##### Barriers and Facilitators

**Fig. 1:**
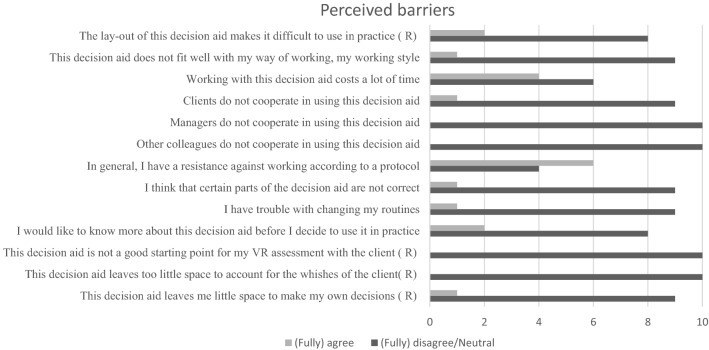
Perceived barriers. (R): item was reversed

Figure 1 shows the barriers and facilitators that VR professionals (n = 10) experienced while using the decision aid. Six of the VR professionals had a general resistance to working according to a protocol. VR professionals did not see the cooperation of colleagues (n = 10 (fully) disagree/neutral), managers (n = 10 (fully) disagree/neutral) or clients (n = 9 (fully) disagree/neutral) as a barrier to using the decision aid in practice. Opinions varied about whether using the decision aid costs a lot of time; (n = 4 (fully) agree, n = 6 (fully) disagree/neutral).

In the interviews, VR professionals did not mention many barriers concerning the use of the decision aid. Barriers that were mentioned, were mainly related to the process that was chosen to implement the decision aid in this feasibility study. VR professionals agreed that this process should be altered when the decision aid is implemented in practice:

Questionnaires were not received on time, also people did not reply. Also, it was more labor intensive, because you had to get the questionnaire, fill in the questionnaire [in Excel]. I did chose to do this myself, because I know that our administration unit is already quite busy. But if you are going to implement this, then I would choose a different way. (VR professional 2)

VR professionals would appreciate it if they could receive the decision aid filled in with the answers of the client. They also mentioned that the current decision aid is slow. A solution participants mentioned, is embedding the decision aid within the IT-systems of the SSI.

##### Attitude

In general, VR professionals had a positive attitude towards the use of the decision aid in practice (n = 9 (fully) agree). The decision aid supports in further professionalizing the work of the VR professional (n = 9 (fully) agree) and it improves the quality of VR care (n = 9 (fully) agree). An overview of all quantitative results for attitude, intention for future use, self-efficacy and knowledge and skills can be found in Tables A2-A5 in Appendix 3.

##### Intention for Future Use

Eight VR professionals had the intention to use (parts of) the decision aid in the future. Six VR professionals indicated that they especially wanted to use the decision aid to get an overview of the RTW barriers of the client.

According to most VR professionals the use of the decision aid should not be mandatory in the future (n = 8 (fully) agree). VR professionals do want to use the decision aid as a supportive tool in their assessments, especially if the barriers for the use of the decision aid mentioned above, are addressed.

I think it can work well in a supportive manner, especially for colleagues who are new, but I don’t think that the decision aid, in this form, should be leading. (VR professional 3).

Indeed, I see it has added value (…), as a supportive tool. In the assessment and in drawing conclusions, and in documentation, and overall. (VR professional 5)

Half of the VR professionals (n = 5) found the decision aid not applicable for all clients. In the questionnaire, VR professionals indicated that they would not recommend applying the decision aid for clients that have insufficient reading skills, have too little knowledge of the Dutch language or clients that are already participating in a VR intervention.

##### Self-efficacy

VR professionals felt confident in using the decision aid for most aspects of their assessment. Examples are: preparing the assessment (n = 9 (fully) agree), getting an overview of the RTW barriers (n = 10 (fully) agree), discussing with the client which barriers play a role in returning to work (n = 10 (fully) agree), and choosing suitable VR interventions (n = 9 (fully) agree).

##### Knowledge and Skills

Although all VR professionals felt they have enough knowledge and skills to use the decision aid in practice, half of them expressed the need for more training. VR professionals would appreciate to have peer-to-peer sessions to discuss the use of the decision aid with their colleagues once they were using the decision aid in practice:

I would like to have something more, maybe some sort of peer-to-peer session, or something onhow to interpret, what do I do with it? Reading the questionnaire was not difficult, and to see where the points requiring attention were, was also not very difficult to me. But then the next step, which parts of the VR need attention, it is almost inside your head, right? The questionnaire sharpens you mind, but maybe there are also other things that should be intervened upon or that we should focus on. Thus, I could benefit from some more training or support with this [working with the decision aid]. (VR professional 1)

## Discussion

Our results showed that, overall, most VR professionals were satisfied with the training and with the decision aid.Also, clients were satisfied with the VR assessments based on the decision aid. Important barriers for implementation could be the general resistance of VR professionals to working according to a protocol (some professional autonomy should remain) and the administrative process that was part of this study. VR professionals had the intention to use the decision aid in the future, but would appreciate additional training.

### Comparison with Literature

The result that VR professionals are positive about using the decision aid for identifying the most important RTW barriers for a client, is in line with earlier studies that developed tools for a similar population (i.e. [12, 13]). Most of the VR professionals in our study want to use (parts of) the decision aid in practice. Other studies also showed that most of the participating rehabilitation professionals were interested in working more evidence-based [18, 19].

We can conclude that some VR professionals perceive insufficient time as a potential barrier for using the decision aid. Earlier studies have also shown that barriers, such as lack of administrative support, insufficient time, and need for training can limit the use of evidence based practices [18]. Our results should, however, be seen in the light of the pilot phase of the decision aid.

Half of our VR professionals indicated a need for more training, especially in periodical discussions about working with the decision aid with colleagues. The importance of this need is supported by a study that showed the limited effect of a single workshop on long-lasting attitude, knowledge and intention to use evidence based medicine [20].

Clients appreciated that the VR professional already had information about their situation and that they had the opportunity to explain their questionnaire answers in the meeting. This way, the decision aid supports including clients in shared decision making. Shared decision making assists empowerment and self-determination in choices and decisions [21, 22]. That clients are more satisfied with VR care if they feel part of the process [23], and that addressing barriers early on makes clients feel more heard [23, 24], strengthens this approach.

### Strengths and Limitations

A strength of this study is that we used comprehensive components of theoretical frameworks ([15, 16]) to test the feasibility of the decision aid, which resulted in detailed information on its use and implementation in practice. Moreover, we included the perspectives of both VR professionals and clients and used a mixed methods design.

A limitation of this study is that selection bias is likely to have occurred in the selection of VR professionals and clients. The VR professionals in this study participated voluntarily and were motivated to use the decision aid in practice. Contrary to our instructions, the VR professionals selected clients (based on, for example, educational level). Therefore, it is likely that the results of this study are more positive than could be expected in a more general population of VR professionals and clients.

A final limitation is the limited length of this study, often only including the first or sometimes second meeting of VR professional and client. Information on VR interventions the VR professional determined using the decision aid áfter the first or second meeting with the client is not included.

### Implications for Practice, Policy and Research

A decision aid can support VR professionals in guiding clients in returning to work. But to improve efficiency, it is essential to embed the decision aid in the IT-systems of the Dutch SSI. However, the digital literacy of the client population should be kept in mind.

Future research should indicate what level of VR interventions is the most feasible for this decision aid and whether the training should be altered to support VR professionals in choosing the correct VR trajectory. Also, the role of contextual factors on the RTW of this group should be addressed. Future research should focus on if and how contextual factors can be implemented in the decision aid.

The protocol should be refined to improve the accessibility of the decision aid, for example by including options to support the client with filling in the questionnaire, or by including criteria to help the VR professional determine for which clients the decision aid is suitable. On top of this, future research should indicate the content and form of additional training in the decision aid.

Future research should indicate if it is feasible to implement the decision aid among a general population of VR professionals and the total population of clients. Future studies should also focus on satisfaction of clients with the decision aid, with the care received by the Dutch SSI, and if the decision aid leads to more clients returning to work.

## Conclusion

This study showed that it is feasible to implement and use the decision aid in practice. Identifying the most important RTW barriers was perceived as more feasible to use in practice than selecting the most suitable VR interventions. Overall, clients and professionals were satisfied with the decision aid, providing good reasons for further implementation.

### Supplementary Information

Below is the link to the electronic supplementary material.

Supplementary file1 (DOCX 15 kb)

Supplementary file2 (DOCX 15 kb)

Supplementary file3 (DOCX 24 kb)

Supplementary file4 (DOCX 110 kb)

## Data Availability

The datasets generated during and/or analyzed during the current study are available from the corresponding author on reasonable request.
