# Supplementary material for: A Decision Aid to Support Vocational Rehabilitation Professionals Offering Tailored Care to Benefit Recipients with a Long-Term Work Disability: A Feasibility Study
Source: J Occup Rehabil. 2023 Apr 10;34(1):128–40. doi: 10.1007/s10926-023-10105-7 (PMC10899301; doi:10.1007/s10926-023-10105-7)
Supplement: Supplementary file 1 — Supplementary file1 (DOCX 15 kb) [file 10926_2023_10105_MOESM1_ESM.docx]

**Appendix 1: Interview guide**

Themes discussed in the interviews with VR professionals and clients:

| **Interviews with VR professionals** |
| --- |
| How the VR professional used the decision aid |
| Barriers and facilitators of using the decision aid |
| Satisfaction with the decision aid |
| Future use of decision aid |
| Barriers and facilitators of implementing the decision aid |
| Influence of the decision aid on evidence-based working |

| **Interviews with clients** |
| --- |
| Satisfaction with the questionnaire of the decision aid |
| Barriers and facilitators for filling in the questionnaire |
| Satisfaction with the process concerning the decision aid |
| Satisfaction with the contact with the VR professional |
| Satisfaction with the decision aid |
| Satisfaction with the suggested VR interventions |
