# Supplementary material for: A Decision Aid to Support Vocational Rehabilitation Professionals Offering Tailored Care to Benefit Recipients with a Long-Term Work Disability: A Feasibility Study
Source: J Occup Rehabil. 2023 Apr 10;34(1):128–40. doi: 10.1007/s10926-023-10105-7 (PMC10899301; doi:10.1007/s10926-023-10105-7)
Supplement: Supplementary file 2 — Supplementary file2 (DOCX 15 kb) [file 10926_2023_10105_MOESM2_ESM.docx]

**Appendix 2 : learning objectives for the training session**

| Table A1: Learning objectives for the training session | |
| --- | --- |
| The professional: | |
| *1* | Knows how the decision aid was developed |
| *2* | Knows on which information the decision aid is based |
| *3* | Knows how the decision aid can be used in the assessment with the client |
| *4* | Knows how the decision aid can be used to recognize RTW barriers of the client |
| *5* | Knows what it means if a factor is a RTW barrier |
| *6* | Knows how the barriers are measured using the questionnaire |
| *7* | Can select the most important RTW barriers in the decision aid |
| *8* | Knows what the VR interventions used in the decision aid entail |
| *9* | Can select the most suitable VR interventions in the decision aid based on the client’s needs |
| *10* | Knows how to document the results of the meeting(s) with the client |
