# Supplementary material for: A Decision Aid to Support Vocational Rehabilitation Professionals Offering Tailored Care to Benefit Recipients with a Long-Term Work Disability: A Feasibility Study
Source: J Occup Rehabil. 2023 Apr 10;34(1):128–40. doi: 10.1007/s10926-023-10105-7 (PMC10899301; doi:10.1007/s10926-023-10105-7)
Supplement: Supplementary file 3 — Supplementary file3 (DOCX 24 kb) [file 10926_2023_10105_MOESM3_ESM.docx]

**Appendix 3: Quantitative results attitude, intentions for future use, self-efficacy and knowledge and skills**

**Table A2: Attitude: (number of participants that fully disagree/fully agree, n=10)**

|  |  | **(Fully) disgree/ neutral** | **(Fully) agree** |
| --- | --- | --- | --- |
| 1 | Dit instrument kan helpen bij het maken van complexe beslissingen  The decision aid can support the VR professional in making complex decisions | 2 | 8 |
| 2 | Het instrument zorgt voor verdere professionalisering van arbeidsdeskundigen / adviseurs intensieve dienstverlening  The decision aid stimulates professionalizing the work of the VR professional | 1 | 9 |
| 3 | Werken volgens het instrument is niet te rigide om toe te passen op de individuele werkzoekende (R)  Working according to the decision aid is not too rigid to apply to the individual client | 3 | 7 |
| 4 | Het instrument zorgt voor een toename in de kwaliteit van de dienstverlening aan de WIA-WGA klant  The decision aid increases the quality of VR care | 2 | 8 |
| 5 | Het instrument kan de AD/AID - klant relatie verbeteren  The decision aid can improve the relationship between the VR professional and the client | 3 | 7 |
| 6 | Het instrument laat genoeg ruimte voor de autonomie van de AD/AID(R)  The instrument allows sufficient space for professional autonomy | 3 | 7 |
| 7 | Werken volgens het instrument belemmert professionals niet in het zich eigen maken van nieuwe inzichten betreffende WIA-WGA klanten(R)  Working according to the decision aid does not hinder VR professionals in gaining new insights about clients | 3 | 7 |
| 8 | Ik kan me vinden in de inhoud van het instrument  I agree with the content of the decision aid | 2 | 8 |
| 9 | Mijn houding ten opzichte van het instrument is positief  I have a positive attitude towards the decision aid | 1 | 9 |

**Table A3: Intention for future use: (number of participants that fully disagree/fully agree, n=10)**

|  | **(Fully) disgree/ neutral** | **(Fully) agree** |
| --- | --- | --- |
| Ik heb de intentie om het instrument, of onderdelen van het instrument, te blijven gebruiken  I intend to use (parts of) the decision aid in the future | 2 | 8 |
| Ik verwacht in de nabije toekomst elementen van het instrument te gebruiken  I expect to use elements of the decision aid in the near future | 3 | 7 |
| Ik heb de intentie om het gehele instrument te blijven gebruiken of te gaan gebruiken  I intent to keep on, or start using the entire whole decision aid | 6 | 4 |
| Het gebruik van het instrument bij begeleiding van de klant zou verplicht moeten worden  It should be mandatory to use the decision aid when supporting clients | 8 | 2 |
| Klanten, die ik begeleid, zouden voordeel hebben van de implementatie van het instrument  Clients that I support, would benefit from implementing the decision aid | 2 | 8 |
| Naar mijn mening, is het instrument voor alle klanten bruikbaar (r)  In my opinion, the decision aid is applicable to all clients | 5 | 5 |
| Het gebruik van het instrument zal leiden tot meer uniformiteit in de begeleiding van WIA-WGA klanten naar werk  Using the decision aid will result in more uniformity in guiding clients to work | 3 | 7 |

**Table A4: Self-efficacy (number of participants that fully disagree/fully agree, n=10)**

|  |  | **(Fully) disgree/ neutral** | **(Fully) agree** |
| --- | --- | --- | --- |
| 1 | Ik voel mij voldoende vaardig om het instrument toe te passen in de praktijk  I feel sufficiently skilled for using the decision aid in practice | 0 | 10 |
| 2 | Het instrument heeft een positieve invloed op de kwaliteit van mijn begeleiding van de klant  The quality of my support to clients is positively influenced by use of the decision aid | 3 | 7 |
| 3 | Het instrument is voor mij bruikbaar om het oriëntatiegesprek met de klant voor te bereiden  The decision aid is useful in preparing the assessment with the client | 1 | 9 |
| 4 | Het instrument is voor mij bruikbaar om de informatie van SMZ in beeld te krijgen  The decision aid is useful in gaining insight in the information from the work capacity assessment | 9 | 1 |
| 5 | Het instrument is voor mij bruikbaar om de belemmerende factoren voor werkhervatting van de klant in beeld te krijgen  The decision aid is useful in providing an overview of the RTW barriers of the client | 0 | 10 |
| 6 | Het instrument is voor mij bruikbaar om in gesprek te gaan met de klant over waar de belemmeringen zitten om terug te keren naar werk  The decision aid is useful for discussing the RTW barriers with the client | 0 | 10 |
| 7 | Het instrument is voor mij bruikbaar om in gesprek te gaan met de klant over welke belemmeringen aangepakt moeten worden  The decision aid is useful for discussing with the client which RTW barriers should be addressed | 2 | 8 |
| 8 | Het instrument is voor mij bruikbaar om samen met de klant passende re-integratiedienstverlening uit te kiezen  The decision aid is useful for choosing suitable VR interventions with the client | 1 | 9 |
| 9 | Het instrument is voor mij bruikbaar om de klant over te dragen aan het re-integratiebureau  The decision aid is useful for the transfer of the client to the reintegration agency | 2 | 8 |
| 10 | Het instrument is voor mij bruikbaar om de klant te monitoren tijdens het re-integratieproces  The decision aid is useful for monitoring the client during the vocational rehabilitation process | 3 | 7 |

**Table A5: Knowledge and skills (number of participants that fully disagree/fully agree, n=10)**

|  |  | **(Fully) disgree/ neutral** | **(Fully) agree** |
| --- | --- | --- | --- |
| 1 | Ik heb voldoende kennis van het instrument om het toe te passen in de praktijk  I have sufficient knowledge of the decision aid to use it in practice | 0 | 10 |
| 2 | Ik heb de vaardigheden om het instrument te kunnen gebruiken  I have the skills to use the decision aid | 0 | 10 |
| 3 | Ik heb geen behoefte aan meer training en oefening in het instrument (r)  I have no need for further training in using the decision aid | 5 | 5 |
| 4 | Ik heb genoeg tijd beschikbaar om met het instrument te leren werken (r)  I have enough time available to learn how to use the decision aid in practice | 6 | 4 |
| 5 | De huidige werkwijze bij UWV laat voldoende ruimte om met het instrument te kunnen werken  The current working process of the SSI leaves enough space for using the decision aid | 6 | 4 |
| 6 | Ik geloof dat het haalbaar is om het instrument in de praktijk te gebruiken  I believe that it is feasible to use the decision aid in practice. | 2 | 8 |

(r) : item was reversed.
