# Supplementary material for: A Decision Aid to Support Vocational Rehabilitation Professionals Offering Tailored Care to Benefit Recipients with a Long-Term Work Disability: A Feasibility Study
Source: J Occup Rehabil. 2023 Apr 10;34(1):128–40. doi: 10.1007/s10926-023-10105-7 (PMC10899301; doi:10.1007/s10926-023-10105-7)
Supplement: Supplementary file 4 — Supplementary file4 (DOCX 110 kb) [file 10926_2023_10105_MOESM4_ESM.docx]

**Supplementary material 1: A Digital Decision Aid to support Vocational Rehabilitation Professionals offering Tailored Care to Work Disability Pension Recipients: An Experimental Study using Case Vignettes**

Christa J.C. de Geus^1,3^, Maaike A. Huysmans^1,3,^ H. Jolanda van Rijssen^1,2,3,^ Trees T. Juurlink^1,3^, Johannes R. Anema^1,3^

^1^ Department of Public and Occupational Health, Amsterdam Public Health Research Institute, Amsterdam UMC, Vrije Universiteit Amsterdam, Van der Boechorststraat 7, NL, 1081 BT Amsterdam, the Netherlands

^2^ Dutch Institute of Employee Benefit Schemes (UWV), Amsterdam, the Netherlands

^3^ Research Centre for Insurance Medicine, AMC-UMCG-VUmc-UWV, Amsterdam, the Netherlands

**Abstract**

**Purpose**: In this experimental study we investigated whether the use of a decision aid during an assessment by a vocational rehabilitation (VR) professional results in an increased agreement with a gold standard of the most important return to work (RTW) barriers and most suitable VR interventions for work disability pension recipients and results in increased confidence of VR professionals in their assessment.

**Methods**: We conducted an experimental study with case vignettes among 23 VR professionals. We used a before-after design to compare whether the assessment of the most important RTW barriers and the most suitable VR interventions were in agreement with a gold standard with and without the use of a decision aid, in which the professionals were trained. Participants were also asked to state their confidence in their assessment of each case.

**Results:** Use of the decision aid significantly improved agreement with the gold standard both in identifying the most important RTW barriers and the most suitable VR interventions. However, use of the decision aid did not have a significant effect on the confidence of professionals.

**Conclusions**: A decision aid can be a promising tool for increasing the quality and conformity of assessments by VR professionals.

**Keywords:** decision aid; labour experts; VR professionals; RTW barriers; VR intervention.

## **Introduction**

People who have been sick listed for a long period often do not return to work [1-3]. Next to receiving financial compensation to limit the loss of income, those who still have the capacity to work may receive interventions aimed at vocational rehabilitation to facilitate their return to work [4,5]. However, even people who receive help with vocational rehabilitation often do not return to work [1,3].

VR professionals are tasked with helping work disability pension recipients (partly) return to work but often experience difficulties to do so. VR interventions are most effective when aimed at specific and personalized RTW barriers [6]. However, it is difficult for VR professionals to assess these often complex situations and determine which intervention could be effective given that they have limited time for assessing their clients’ situations [7] and that clients often have multiple problems that can inhibit them from returning to work. There is also a lack of evidence on which factors play the most important roles in RTW [8], as well as a lack of knowledge of which VR interventions are most effective for positively influencing these RTW factors [9]. There are currently only a few evidence-based tools that can support VR professionals in their assessments, such as the WHODAS 2.0 [10], the Work-ability Support Scale [11], and the Work Needs Assessment [12].

VR professionals could be supported in their assessments of important RTW barriers and suitable VR interventions by a decision aid. Decision aids are meant to complement the expertise, know-how, and experience of a professional by illustrating possible outcomes [13]. Earlier research has shown that decision aids and educational programmes can be effective at increasing the quality of health care [14], knowledge of guidelines and performance of occupational health professionals [15,16]. Earlier on, we developed a digital decision aid for VR professionals based on the results of a Delphi study [17]. In the Delphi study, experts determined which RTW barriers were most important for the vocational rehabilitation of disability pension recipients and which VR interventions were suitable for influencing these RTW barriers to facilitate return to work. The results were used to develop the decision aid for VR professionals supporting (partial) work disability pension recipients. The decision aid gives an overview of the RTW barriers and facilitators of work disability pension recipients and suggests suitable VR interventions. The aim of the decision aid is to increase evidence-based assessment and decrease variation in assessment among VR professionals. VR professionals received a training session on the use of the decision aid before using it.

The objective of the present study was to investigate whether the use of the digital decision aid by VR professionals resulted in increased agreement with a gold standard for most important RTW barriers and most suitable VR interventions for work disability pension recipients. We used increased agreement with the gold standard as a proxy for more customized and evidence-based services, and thus for more effective RTW support for people with a work disability. An additional objective was to investigate whether using the decision aid increased VR professionals’ confidence in identifying the most important RTW barriers and the most suitable VR interventions to influence RTW barriers.

## **Methods**

Context:

People in the Netherlands who have been on sick leave for two years can apply for a work disability pension at the Dutch Social Security Institute (SSI) according to the Act on Work and Income According to Work Capacity (WIA). Work disability pension recipients sometimes still have some capacity to work. In such cases, they receive only a partial work disability pension and are expected to generate part of their income. These people receive help from VR professionals employed at the SSI with their return to work. VR professionals assess clients face-to-face to determine which RTW barriers need to be addressed to facilitate RTW with a VR intervention. Based on this assessment, a rehabilitation plan is developed, and the work disability pension recipient receives a VR intervention, often from a third party. The progress of the person is monitored by the VR professional.

***Study Design***

An experimental study was conducted among 23 VR professionals. For this study, six pairs of matching textual case vignettes were developed based on real client records. The VR professionals completed two questionnaires concerning the case vignettes. In the baseline questionnaire, participants had to determine 1) the most important RTW barriers and 2) the most suitable VR interventions for the six case vignettes *without* the use of a decision aid (three case vignettes for assessing RTW barriers and three case vignettes for assessing suitable VR interventions). Participants were also asked about their confidence in their assessments. They then received a training session on the use of the decision aid. After the training session, participants received a follow-up questionnaire asking them to once again determine the most important RTW barriers and most suitable VR interventions for six matching case vignettes, this time *with* the use of the decision aid. Participants were also asked to again rate their confidence in their assessment.

The importance of RTW barriers and most suitable VR interventions were determined according to a gold standard we developed. The gold standard was developed based on consensus of three experts. We then determined the level of agreement between the answers of study participants and the gold standard. We also measured the differences in the confidence of the participant in their assessment with and without the decision aid.

***Study Population***

The study population consisted of non-medical VR professionals working at the Dutch SSI who were tasked with supporting partial work disability pension recipients in their vocational rehabilitation. VR professionals were invited to participate in this study via the SSI’s internal newsletter and selected using snowball sampling. People were eligible for participation in this study if they met the following criteria: non-medical VR professional employed at the Dutch SSI; at least six months of experience with supporting work disability pension recipients with remaining work capacity; and experience with face-to-face contact with disability pension recipients who were recently granted a work disability pension.

Participation in this study was voluntary. All participants signed an informed consent form and data were anonymized. This study was approved by the Medical Ethics Committee of Amsterdam UMC, VU University Medical Centre Amsterdam (2021.0406). The committee declared that no comprehensive ethical approval was needed for this study.

***Decision Aid***

The aim of the digital decision aid is to increase evidence-based work and the delivery of more tailored care among VR professionals by helping to 1) identify the most important RTW barriers and 2) determine the most suitable VR intervention(s) for a work disability pension recipient. The content of the decision aid was developed based on a Delphi Study [17] in which the most important RTW factors and suitable VR interventions were determined. The decision aid was developed in close collaboration with important stakeholders with experience with decision aids and with the vocational rehabilitation of work disability pension recipient. The result was a digital tool in Microsoft Excel for VR professionals, an overview of which can be found in table 1.

[ insert table 1]

The decision aid consists of a questionnaire that is filled in by the disability pension recipient to determine important RTW barriers and RTW facilitators. Based on answers in the questionnaire, the decision aid identifies which factors are RTW barriers and which factors are RTW facilitators using a traffic light code system. RTW barriers are shown as red factors, possible RTW barriers are shown as orange factors, and RTW facilitators are shown as green factors. The decision aid is used for structuring the VR professional’s first meeting with a client. In this meeting, the VR professional discusses the RTW barriers from the questionnaire with the client. Together they choose the RTW barriers that will be targeted with one or more VR interventions. Based on the chosen RTW barriers, the decision aid suggests VR interventions tailored to these barriers. One VR intervention can often target several different RTW barriers. The decision aid ranks the suitable VR interventions based on 1) the extent to which the VR intervention is suitable to target a certain RTW barrier and 2) the number of RTW barriers the VR intervention targets. The VR professional discusses which VR interventions are most suitable with their client, and based on the outcomes of this meeting and the professional opinion of the VR professional, a tailored VR programme is developed consisting of one or more VR interventions. The VR programme is offered by a third party. The progress of the client is supervised by the VR professional from the SSI.

The decision aid training session introduced the VR professionals to the tool and demonstrated how the decision aid should be used in the daily practice. All training sessions were organized and conducted by at least two members of the research team. The training sessions were conducted online using Microsoft Teams, lasted two hours, and consisted of several elements. The sessions started by explaining how the decision aid had been developed and then explained the different elements and how to use the decision aid in practice. Thereafter, participants were given the opportunity to test the decision aid using examples of cases they had recently assessed. Finally, participants practiced using the decision aid with a case provided by the research team. The training sessions concluded with the participants reflecting on the use of the decision aid.

***Case Vignettes***

Case vignettes were developed in cooperation with three VR professionals with extensive experience in the vocational rehabilitation of work disability pension recipients. The case vignettes were based on types of clients that are typically seen in this target group. We made six pairs of case vignettes: three for determining barriers and three for determining interventions. A pair consisted of cases with similar barriers for RTW: physical or mental limitations, level of work disability and amount of pension, educational level, and work history. To prevent participants from recognizing a case and answering the questions the same way as without the decision aid, the cases presented varied characteristics (such as name, gender, living area, family circumstances, and hobbies) within each pair. Table 2 shows a summary of the twelve case vignettes and an example of a case vignette. The barriers were also varied among the cases in such a way that most of the barriers used in the decision aid were mentioned in the cases at least once. The pairs of cases were then divided so that one case vignette was in the baseline questionnaire and the other matching case vignette was in the follow-up questionnaire.

[insert table 2]

The vignettes included a short overview of the background information of the client: name, age, profession, level of education, work history, level of work disability, and pension and remaining earning capacity. The vignettes also included a short overview of the functional limitations that had been established by an insurance physician during the work disability assessment, followed by an overview of information on the daily living situation of the client. RTW barriers of the client in the case were mentioned in the text of the vignettes.

***Baseline Questionnaire***

The baseline questionnaire consisted of three sections. The participants first filled out a section with background information, including age, gender, work location, type of occupation, and years of experience with the vocational rehabilitation of this group. The second section focused on RTW factors. In this section, three case vignettes were shown. For each vignette, participants were asked—without the support of the decision aid—to indicate which factors were a barrier or were not a barrier for the RTW of the client. Participants also had the option of answering “cannot determine.” Subsequently, participants were asked to indicate which three RTW barriers were the most important to target in a VR intervention. After each case vignette, participants were asked to indicate how confident they were in their assessment on a scale of 1–10. The third and last section focused on VR interventions. In this section, three other case vignettes were shown. For these case vignettes, the three most obstructing RTW barriers were given. Based on this information, participants were asked to assess if a type of VR intervention was appropriate or not for the worker in the case vignette. Participants also again had the option of answering “cannot determine.” Participants were next asked which three types of VR intervention were most suitable for the client in the case vignette to target the specific RTW barriers of that client. Participants had the option to explain their answer in an open text field. Finally, after each case vignette, participants were asked to indicate their level of confidence with their assessment on a scale of 1–10.

***Follow-up Questionnaire***

After the training session, participants filled in a follow-up questionnaire consisting of the second and third sections of the baseline questionnaire. In the follow-up questionnaire, six new case vignettes with the outcomes of the decision aid (including the answers of the client on the questionnaire of the decision aid) were presented.

***Outcomes***

The primary outcome of this study was the difference in agreement with a gold standard on the most important RTW barriers and the most suitable VR interventions when using the decision aid compared to not using the decision aid. The secondary outcome of the study related to the confidence of professionals in their own assessment.

*Primary outcome*

To develop a gold standard for measuring the primary outcome, three experts were recruited: an experienced insurance physician (co-author: JRA) and two experienced VR professionals (SM and MH). These experts had previously been involved in the development of the decision aid. After being trained in the use of the decision aid, the experts scored the case vignettes independently. They then received the outcomes of the decision aid for all case vignettes. In an online consensus meeting, the differences in scores were discussed for the cases in which agreement had not yet been reached. At the end of the online meeting, consensus was reached for which RTW barriers and which VR interventions were most important for each case vignette and thus part of the gold standard.

Subsequently, in the evaluation of study outcomes, participants were given one point for each RTW barrier or VR intervention they mentioned that was in agreement with the gold standard. For each case vignette, a participant could thus receive up to three points, for a total of nine points for determining the important RTW barriers and determining the most suitable VR interventions, as detailed below (Table 3). A higher score indicated greater agreement with the gold standard.

*[insert table 3]*

*Secondary Outcome*

The secondary outcome of this study related to participants’ confidence in their own assessments. This was measured with one question after each case vignette: “On a scale of 1 (very low) to 10 (very high) can you indicate how high your confidence is that you selected (a) the most important RTW barriers or (b) the most suitable VR interventions to increase the chances on work?” Participants’ confidence for the case vignettes assessed with the decision aid was compared to their confidence for the case vignettes which were assessed without the decision aid.

***Statistical Analysis***

Descriptive statistics were used to report on the demographic and professional characteristics of the participants. We used linear mixed models (LMM) to test agreement with the gold standard without the use of the decision aid and with the use of the decision aid. We first evaluated the efficacy of the use of a decision aid in determining the most important RTW barriers (Model 1) and then for determining the most suitable VR intervention (Model 2).

Linear mixed models were also used to evaluate the influence of the decision aid on the VR professionals’ confidence in assessment.

In our models, use of the decision aid was added to the model as a fixed effect. The participant ID and number of the case vignette were specified as random effects. For both outcomes, we calculated the Wald confidence interval of the effect at the 95% level. We obtained the p-value of the main effects using the type-II ANOVA test implemented in the R package “car.” In both analyses, we considered the significance level to be alpha=0.05.

## **Results**

Twenty-three VR professionals participated in this study. Table 4 presents the demographic and professional characteristics of the participants. Most of the VR professionals were older than 45 years (69.5%) and the majority were female (83%).

*[insert table 4]*

***Practice Variation in Assessment with and without the use of the Decision Aid***

For each case, the average agreement with the gold standard is given in Figure 1 (0=no agreement with gold standard, 3=maximum agreement with gold standard) and Table 5. For all case pairs, except case pair 6, the average agreement with the gold standard was higher for the case vignette evaluated using the decision aid compared to not using the decision aid.

*[insert figure 1]*

*[insert table 5]*

The use of the decision aid was significantly associated with a higher score on the gold standard for both determining the most important RTW barriers (Model 1, case pair 1–3, B=0.57, 95%CI (0.33-0.80)) and for determining the most suitable VR interventions (Model 2, case pair 4-6, B=0.23, 95%CI (0.01-0.45)). The results of the LMM analyses are summarized in Table 6.

*[insert table 6]*

***Confidence in Assessment with and without the Decision Aid***

The confidence of VR professionals was higher for all cases with the use of the decision aid, but this increase in confidence was not significant (see Table 5). The average confidence ratings that VR professionals gave their own assessments with and without the use of the decision aid for each case are presented in Figure 2 and table 5.

*[insert figure 2]*

The use of the decision aid was determined not to have a significant effect on the confidence of participants, neither in determining important RTW barriers (Model 1, case pair 1–3, (B=0.26, 95% CI (-0.00-0.05))) nor in determining the most suitable VR interventions (Model 2, case pair 4–6, (B=0.20, 95% CI (-0.01-0.42))) (Table 6).

**Discussion**

The aim of the present study was to investigate whether the use of a digital decision aid by VR professionals would result in increased agreement with a gold standard regarding the most important RTW barriers and the most suitable VR interventions for people receiving a work disability pension. We used increased agreement with the gold standard as a proxy for the delivery of more customized and evidence-based services. And thus, for better tailored and evidence-based support to increase their chances for acquiring paid employment despite having a work disability. This study showed that using the decision aid significantly increased agreement with the gold standard, which implies that using the decision aid helps VR professionals to be more evidence-based in their work and helps to reduce practice variation between VR professionals. Using the decision aid did not, however, increase the confidence of the VR professionals in their own assessments.

A previous study by Schouten et al. [12] showed that training in using a tool and actual use of a tool aimed at identifying work support needs, did indeed help to identify work support needs of clients, but did not improve the consistency of the professionals’ assessment of suitable VR interventions, as we found in our study. This may have been due to the fact that the tool of Schouten et al. only helped to identify relevant RTW factors, but did not suggest relevant VR interventions as the decision aid did in the present study. In our decision aid these steps are both included and linked, which might explain the increased consistency we found between the professionals’ assessment of suitable VR interventions. In addition, our study showed that when professionals are trained in using a tool and are prompted to actually use it, professionals seem inclined to incorporate the tool in their daily practice. This implies that a tool can contribute to the improvement of evidence-based practice among professionals. On the long-term, however, the effects are unclear. Studies showed that educational programmes can be effective in increasing knowledge of guidelines, guideline adherence, and performance (15,16), but also that these programmes are not always effective in increasing guideline adherence on the long-term (18,19). An earlier study in insurance medicine showed that a workshop did increase skills in evidence-based practice measured after three months, but did not increase knowledge on evidence-based practice after three months [18]. Adding a tool to the training, as we did in the current study, might contribute to evidence-based working on the long-term, but additional research is needed to confirm this.

The significant overall effect of the decision aid on agreement with the gold standard for the RTW factors could be weighted by including case 3 in the analysis. The difference in average agreement with the gold standard with (M=1.3 (SD=0.70)) and without (M=0.22 (SD=0.42)) using the decision aid seemed to be especially large for this case. Therefore, we performed a sensitivity analysis by removing case 3 from the model. This indeed decreased the overall effect, but agreement with the gold standard was still significantly larger when using the decision aid (B=0.30 95%CI (0.01-0.60), p=0.04)).

***Confidence in Assessment***

The results of this study showed that using the decision aid did not increase VR professionals’ confidence in their own assessments. Confidence was already quite high without the decision aid, which may have left little room for improvement. Yet, we do see a small improvement in the level of confidence, and thus it may also be possible that the number of participants was too small to detect an effect on this secondary outcome. Another explanation might be that the outcome of the decision aid was in line with their own thoughts. This possibility would be in line with the results of a similar study among insurance physicians, in which the researchers found that if the decision aid showed the same outcomes as the insurance physician’s own assessment there was no significant change in confidence. In that study, only in cases where the decision aid showed a different outcome than the physician’s own assessment was a significant decrease in confidence found [13].

***Strengths and Limitations***

In the present study, we tested the efficacy of using a decision aid using case vignettes on paper. Despite the limitation of case vignettes that professionals have to base their assessment on a relatively short description on paper and cannot ask questions or discuss factors with the client, as in real practice [12], the use of case vignettes in this study has several advantages. An important strength of using case vignettes on paper or video is that all VR professionals had the same information on each case. Earlier studies (e.g., [12]) used actors to play cases to simulate real practice. However, there can be a variation in the performances and reactions of actors, which can lead to a higher variation in assessment of the VR professional. Another advantage of using case vignettes is that no actual clients, who are already in a vulnerable position, needed to be burdened by being assessed by multiple VR professionals.

An additional strength of this study was that we included VR professionals from different offices of the Dutch SSI. Each office has their own work procedures and claimants’ populations [13] and by including professionals from different offices we could test the efficacy of the decision aid for a wide variety of VR professionals with practice variation due to different work procedures and claimant’s populations. A final strength is that we were able, due the research design, to test separately the efficacy of a decision aid on RTW factors and VR interventions. Earlier studies focussed on tools that only include RTW factors (e.g., 12), and therefore did not evaluate the efficacy on VR interventions to facilitate return to work.

One of the limitations of this study was the use of a gold standard based on consensus among experts. Due to a lack of professional guidelines, we could not base the gold standard on more objective data. We countered this limitation by asking three experts to participate, thus making sure the gold standard is inter-subjective [19]. Another limitation of this study is that we included 12 case vignettes to test our decision aid. Participants indicated that they had trouble with concentrating on this many cases. This may have led that the last cases were not as thoroughly assessed by the participants as the first cases. An additional limitation is that VR professionals participated voluntarily, which led to a potential selection bias. It is likely that the participants of our study are more interested in working according to a guideline and working with evidence-based tools.

***Implications for Research and Practice***

With this experimental study we showed that using a decision aid helps VR professionals work in ways that are more evidence-based and that reduce the practice variation. Our decision aid is, to our knowledge, the first tool that not only helps to identify relevant RTW barriers but also suggests suitable VR interventions. Although VR professionals recognize the value of evidence-based practice, studies show that there are many barriers to the use of evidence-based knowledge in daily practice [20]. These barriers can include weak administrative support, inadequate funding, too little time, lack of technical skills, and a lack of knowledge [20,21]. Therefore, a feasibility study should be done to test whether the use of this decision aid would be feasible practice and whether the decision aid would increase the quality of the VR services. Another important step would be to evaluate its effectiveness in real practice in a randomized controlled trial, to see whether using the decision aid would lead to greater agreement among VR professionals and to a greater rate of RTW success among disability pension recipients who received VR services.

## **Conclusions**

The present study showed that using a digital decision aid increased the agreement of professionals with a gold standard when assessing the most important RTW barriers and the most suitable VR interventions. This implies that a decision aid can be an effective tool for increasing evidence-based practice and reducing practice variation among VR professionals. Future studies should test whether the use of such a decision aid would be feasible in real life practice and whether using the decision aid would indeed increase RTW success among disability pension recipients.

## **References**

1. Henderson M, Glozier N, Elliott KH. Long term sickness absence. British Medical Journal Publishing Group; 2005. p. 802-803.

2. Waddell G, Burton AK, Kendall NA. Vocational rehabilitation–what works, for whom, and when?(Report for the Vocational Rehabilitation Task Group). TSO; 2008.

3. Louwerse I, Huysmans MA, van Rijssen HJ, et al. Characteristics of individuals receiving disability benefits in the Netherlands and predictors of leaving the disability benefit scheme: a retrospective cohort study with five-year follow-up. BMC Public Health. 2018;18(1):1-12.

4. Anner J, Schwegler U, Kunz R, et al. Evaluation of work disability and the international classification of functioning, disability and health: what to expect and what not. BMC Public Health. 2012;12(1):1-8.

5. de boer WEL. Quality of evaluation of work disability. 2010.

6. Bosselaar H, Maurits E, Molenaar-Cox P, et al. Multiproblematiek bij cliënten, verslag van een verkinning in relatie tot (arbeids)participatie. 2010.

7. Brongers KA, Cornelius B, van der Klink JJ, et al. Development and evaluation of a strength-based method to promote employment of work-disability benefit recipients with multiple problems: a feasibility study. BMC Public Health. 2020;20(1):1-10.

8. Gragnano A, Negrini A, Miglioretti M, et al. Common psychosocial factors predicting return to work after common mental disorders, cardiovascular diseases, and cancers: a review of reviews supporting a cross-disease approach. Journal of occupational rehabilitation. 2018;28(2):215-231.

9. Vogel N, Schandelmaier S, Zumbrunn T, et al. Return-to-work coordination programmes for improving return to work in workers on sick leave. Cochrane Database Syst Rev. 2017 Mar 30;3:CD011618.

10. Üstün TB, Chatterji S, Kostanjsek N, et al. Developing the World Health Organization disability assessment schedule 2.0. Bulletin of the World Health Organization. 2010;88:815-823.

11. Fadyl JK, McPherson KM, Schlüter PJ, et al. Development of a new tool to evaluate work support needs and guide vocational rehabilitation: the Work-ability Support Scale (WSS). Disability and rehabilitation. 2015;37(3):247-258.

12. Schouten MJ, Nieuwenhuijsen K, Wind H, et al. Usability and consistency in findings of the work support needs assessment tool. Work. 2021;68(1):243-253.

13. Louwerse I, Huysmans M, van Rijssen H, et al. Use of a Decision Support Tool on Prognosis of Work Ability in Work Disability Assessments: An Experimental Study Among Insurance Physicians. Journal of Occupational Rehabilitation. 2021;31(1):185-196.

14. Teich JM, Merchia PR, Schmiz JL, et al. Effects of computerized physician order entry on prescribing practices. Archives of internal medicine. 2000;160(18):2741-2747.

15. Zwerver F, Schellart AJ, Knol DL, et al. An implementation strategy to improve the guideline adherence of insurance physicians: an experiment in a controlled setting. Implementation Science. 2011;6(1):1-10.

16. Smits P, Verbeek J, Van Dijk F, et al. Evaluation of a postgraduate educational programme for occupational physicians on work rehabilitation guidelines for patients with low back pain. Occupational and environmental medicine. 2000;57(9):645-646.

17. de Geus CJC, Huysmans MA, van Rijssen HJ, et al. Return to work factors and vocational rehabilitation interventions for long-term, partially disabled workers: a modified Delphi study among vocational rehabilitation professionals. BMC Public Health. 2022 May 2;22(1):875.

18. Kok R, Hoving JL, Verbeek JH, et al. Evaluation of a workshop on evidence-based medicine for social insurance physicians. Occupational medicine. 2008;58(2):83-87.

19. Schellart AM, Zwerver F, Knol D, et al. Development and reliability of performance indicators for measuring adherence to a guideline for depression by insurance physicians. Disability and Rehabilitation. 2011;33(25-26):2535-2543.

20. Fitzgerald S, Leahy MJ, Kang H-J, et al. Perceived preparedness to implement evidence-based practice by certified rehabilitation counselors: A qualitative content analysis. Rehabilitation Counseling Bulletin. 2017;60(4):203-214.

21. Pfaller JS, Tu W-M, Morrison B, et al. Social-cognitive predictors of readiness to use evidence-based practice: A survey of community-based rehabilitation practitioners. Rehabilitation Counseling Bulletin. 2016;60(1):7-15.

**Figures**

Figure 1: Average agreement with gold standard per case. For case pairs 1–3, participants determined the most important RTW barriers. For case pairs 4–6, participants determined the most suitable VR interventions.

Figure 2: Average confidence in assessment per case.

**Tables**

**Table 1: contents of the Decision aid**

| **RTW factors** | **VR interventions** |
| --- | --- |
| **Work and finding work** |  |
| **Job self-efficacy** | 1. Improving self-image and self-knowledge |
|  | 1. Improving vitality and physical resilience |
|  | 1. Identifying what the disabled worker can do in terms of work |
|  | 1. Increasing psychological resilience |
|  | 1. Increasing work experience |
| **Job application skills** | 1. Improving skills and helping to apply for jobs |
|  |  |
| **Job search behaviour** | 1. Improving skills and helping to apply for jobs |
|  | 2. Identifying what the disabled worker can do in terms of work |
|  |  |
| **Knowledge of the labour market** | 1. Identifying what the disabled worker can do in terms of work |
|  |  |
| **Willingness to make concessions** | 1. Informing the disabled worker about the disability benefit or the re-integration process |
|  | 1. Identifying what the disabled worker can do in terms of work |
|  |  |
| **Diplomas** | 1. Training |
|  |  |
| **Proficiency in Dutch language** | 1. Language course |
|  |  |
| **Transportation** | 1. Facilitating transportation |
|  |  |
| **Personal Factors** |  |
|  |  |
| **Importance of work** | 1. Improving societal participation |
|  | 1. Improving self-image and self-knowledge |
|  | 1. Increasing work experience |
|  | 2. Increasing motivation |
|  | 2. Identifying what the disabled worker wants to do in terms of work |
|  | 3. Identifying what the disabled worker can do in terms of work |
|  |  |
| **Motivation to RTW** | 1. Increasing motivation |
|  | 2. Improving self-image and self-knowledge |
|  | 3. Improving vitality and physical resilience |
|  | 3. Increasing work experience |
|  | 4. Improving societal participation |
|  | 4. Identifying what the disabled worker wants to do in terms of work |
|  |  |
| **RTW expectations** | 1. Improving societal participation |
|  | 1. Improving self-image and self-knowledge |
|  | 2. Identifying what the disabled worker wants to do in terms of work |
|  | 2. Identifying what the disabled worker can do in terms of work |
|  |  |
| **RTW self-efficacy** | 1. Improving self-image and self-knowledge |
|  | 1. Improving vitality and physical resilience |
|  | 1. Identifying what the disabled worker can do in terms of work |
|  | 1. Increasing psychological resilience |
|  | 1. Increasing work experience |
|  |  |
| **Coping** | 1. Improving societal participation |
|  | 1. Increasing psychological resilience |
|  | 2. Improving self-image and self-knowledge |
|  | 3. Increasing motivation |
|  | 3. Improving vitality and physical resilience |
|  | 4. Cognitive behavioural therapy |
|  |  |
| **Fear avoidance behaviour** | 1. Improving self-image and self-knowledge |
|  |  |
| **Pain** | 1. Continuing VR interventions as much as possible |
|  |  |
| **Self-esteem** | 1. Improving self-image and self-knowledge |
|  | 2. Increasing psychological resilience |
|  |  |
| **Work functioning** |  |
|  |  |
| **Work ability** | 1 Improving vitality and physical resilience |
|  | 1. Identifying what the disabled worker can do in terms of work |
|  | 1. Increasing work experience |
|  | 2. Improving societal participation |
|  | 2. Improving self-image and self-knowledge |
|  | 2. Increasing psychological resilience |
|  |  |
| **Societal participation** | 1. Improving societal participation |
|  | 2. Increasing motivation |
|  | 2. Increasing work experience |
|  | 3. Improving self-image and self-knowledge |
|  | 3. Strengthening employee skills |
|  |  |
| **Work-life balance** | 1. Improving self-image and self-knowledge |
|  | 2. Improving societal participation |
|  |  |
| **Self-sufficiency** | 1. Improving societal participation |
|  |  |
| **Employee skills** | 1. Strengthening employee skills |
|  |  |
| **External factors** |  |
|  |  |
| **Caring for children** | 1. Referral to services offered by other organizations |
|  |  |
| **Family issues** | 1. Referral to services offered by other organizations |
|  | 2. Cognitive behavioural therapy |
|  | 3. Increasing psychological resilience |
|  | 4. Improving self-image and self-knowledge |
|  | 5. Multidisciplinary interventions |
|  |  |
| **Recent life events** | 1. Increasing psychological resilience |
|  | 2. Referral to services offered by other organizations |
|  | 3. Cognitive behavioural therapy |
|  |  |
| **Social network** | 1. Improving societal participation |
|  |  |
| **Objection or appeal to decision for disability pension** | 1. Continuing VR interventions as much as possible |
|  |  |
| **Secure housing** | 1. Referral to services offered by other organizations |
|  |  |
| **Financial problems** | 1. Referral to services offered by other organizations |
|  |  |
| **Treatment** | 1. Continuing VR interventions as much as possible |
|  |  |
| **Alcohol / substance abuse** | 1. Referral to services offered by other organizations |
|  | 1. Explore feasibility of VR interventions |
|  | 1. RTW services specialized in substance abuse |
|  |  |
| **Health** |  |
|  |  |
| **Perceived general health** | 1. Improving self-image and self-knowledge |
|  | 1. Improving vitality and physical resilience |
|  | 2. Increasing psychological resilience |
|  |  |
| **Quality of life** | 1. Improving self-image and self-knowledge |
|  |  |
| **Unhealthy lifestyle** | 1. Improving vitality and physical resilience |

| **Table 2: overview of case vignettes** | | | | |
| --- | --- | --- | --- | --- |
|  |  | **General information: Sex. age, educational level, profession, work history** | **Percentage of work disability (%)**  **Prognosis** | **Gold standard** |
| **Factors** |  |  |  |  |
| Case pair 1:  RTW barriers suggested by the decision aid:  Likely RTW barriers:   - Social support - Importance of work - Motivation to RTW - Work ability - Knowledge of the labour market - RTW expectations   Possible RTW barriers:   - Perceived general health - Coping | Without decision aid | - Man (age 57), - Primary school - Steel worker - Different functions in the steel industry | 61%  Symptoms will not improve | - Knowledge of the labour market - Importance of work - Motivation to RTW |
|  | With decision aid | - Female (age 59) - Domestic school - Cleaning lady in elderly care - Cleaning lady in elderly care for the last 12 years, and before at other companies | 59%  Symptoms will not improve | - Knowledge of the labour market - Importance of work - Motivation |
| Case pair 2:  RTW barriers suggested by the decision aid:  Likely RTW barriers:   - RTW expectations - Coping - Fear avoidance behaviour - Self-esteem - Work life balance - Willing to make concessions   Possible RTW barriers:   - Quality of life - Work ability | Without decision aid | - Male (age 34) - Law enforcement (bachelor’s and master’s degrees) - Criminal lawyer for 5 years | 45%  Not mentioned | - Self-esteem - Coping - Fear avoidance behaviour |
|  | With decision aid | - Female (age 32) - Auditor, university post-master - Accountant - Accountant for 8 years at a large accountancy firm | 41%  Not mentioned | - Self-esteem - Coping - Fear avoidance behaviour |
| Case pair 3:  RTW barriers suggested by the decision aid:  Likely RTW barriers:   - Unhealthy lifestyle - Family issues - Financial problems - RTW self-efficacy - Pain - Willingness to make concessions   Possible RTW barriers:   - Fear avoidance behaviour - Transportation | Without decision aid | - Female (age 38) - MBO 3 Allround Haarstylist - Hairdresser - Hairdresser for 18 years | 65%  Symptoms will not improve | - Willingness to make concessions - RTW self-efficacy - Unhealthy lifestyle |
|  | With decision aid | - Male (age 40) - MBO 3 Allround Carpenter - Carpenter - Carpenter for 22 years in a small carpentry company | 63%  Symptoms will not improve | - Financial problems - RTW self-efficacy - Unhealthy lifestyle |
| **Interventions** |  |  |  |  |
| Case pair 4:  Suitable VR interventions suggested by the decision aid:   - Increasing vitality and physical resilience - Increasing psychological resilience - Referral to services offered by other organizations - Cognitive behavioural therapy - Identifying what the disabled worker can do in terms of work - Increasing work experience - Improving societal participation | Without decision aid | - Male (age 38) - MBO 4 - Car technology - Mechanic - Car mechanic for 3 years at a BMW Previously worked as a (apprentice) mechanic at 2 other garages. | 50%  Symptoms will not improve | - Increasing psychological resilience - Increasing vitality and physical resilience - Identifying what the disabled worker can do in terms of work |
|  | With decision aid | - Female (age 34) - MBO 3 - Nursing / care - Nurse / caregiver - For the last 5 years she was working in a nursing home. Before that she worked for 3 different employers within the healthcare sector. | 47%  Symptoms will not improve | - Increasing psychological resilience - Identifying what the disabled worker can do in terms of work - Increasing vitality and physical resilience |
| Case pair 5:  Suitable VR interventions suggested by the decision aid:   - Improving self-image and self-knowledge - Continuing VR interventions as much as possible Improving skills and helping with applying for a job - Identifying what the disabled worker can do in terms of work | Without decision aid | - Female (age 29) - Havo, HBO management and economy - Administrative assistant - Administrative assistant at a financial administration office for the last 7 years | 39%  Not mentioned | - Improving self-image and self-knowledge - Continuing RTW interventions as much as possible - Improving skills and helping with applying for a job - Identifying what the disabled worker can do in terms of work |
|  | With decision aid | - Male (age 29) - HBO - Facility management - Facility manager at a school community for the last 5 years | 37%  Not mentioned | - Improving self-image and self-knowledge - Continuing RTW interventions as much as possible - Improving skills and helping with applying for a job - Identifying what the disabled worker can do in terms of work |
| Case pair 6:  Suitable VR interventions suggested by the decision aid:   - Increasing vitality and physical resilience - Increasing motivation - Improving self-image and self-knowledge - Increasing work experience - Improving societal participation - Identifying what the disabled worker wants to do in terms of work - Strengthening employee skills | Without decision aid | - Male (age 37) - HBO - ICT - IT specialist - Started as a junior IT specialist at an international company. Worked as a senior IT specialist at 2 employers in the same sector the past 6 years. | 51.25%  Not mentioned | - Improving societal participation - Improving self-image and self-knowledge - Increasing psychological resilience |
|  | With decision aid | - Female (age 34) - HBO Human resource management - Recruiter - Worked as a recruiter in the industrial sector for 4 years. Previously worked as a recruiter at Randstad. | 54.76%  Not mentioned | - Improving self-image and self-knowledge - Improving societal participation - Increasing vitality and physical resilience |

| Table 3: Calculating points based on the gold standard |
| --- |

|  | Number of case vignettes | Maximum points per case vignette | Total maximum points |
| --- | --- | --- | --- |
| Baseline questionnaire (without decision aid, without training) |  |  |  |
| Determining most important RTW barriers (Cases 1, 2, 3) | 3 | 3 | 9 |
| Determining most suitable VR interventions (Cases 4,5,6) | 3 | 3 | 9 |
| Follow-up questionnaire (with decision aid, with training) |  |  |  |
| Determining most important RTW barriers (Matching Cases 1, 2, 3) | 3 | 3 | 9 |
| Determining most suitable VR interventions (Matching Cases 4, 5, 6) | 3 | 3 | 9 |

Table 4: Demographic and professional characteristics of the participants

|  | **N** | **%** | **Mean (SD)** |
| --- | --- | --- | --- |
| Gender |  |  |  |
| *Male* | 4 | 17.4% |  |
| *Female* | 19 | 82.6% |  |
| Age |  |  | 48.8 (9.8) |
| <35 | 2 | 8.7% |  |
| 35–44 | 5 | 21.7% |  |
| 45–54 | 9 | 39.1% |  |
| 55+ | 7 | 30.4% |  |
| Years of work experience with work disability pension recipients |  |  | 7.9 (5.8) (1-23) |

Table 5: descriptive statistics for agreement with gold standard and confidence in assessment

|  | **Agreement with gold standard** | | **Confidence in assessment** | |
| --- | --- | --- | --- | --- |
|  | **Without decision aid**  **Mean (SD)** | **With decision aid**  **Mean (SD)** | **Without decision aid**  **Mean (SD)** | **With decision aid**  **Mean (SD)** |
| **Case pairs on return to work barriers** |  |  |  |  |
| Case pair 1 | 1.74 (0.75) | 1.87 (0.46) | 7.70 (0.88) | 7.78 (0.95) |
| Case pair 2 | 1.04 (0.77) | 1.52 (0.85) | 7.39 (0.89) | 7.91 (0.9) |
| Case pair 3 | 0.22 (0.42) | 1.3 (0.70) | 7.09 (1.20) | 7.26 (1.05) |
| Average | 1.00 (0.91) | 1.57 (0.72) | 7.39 (1.02) | 7.65 (1.00) |
| **Case pairs on vocational rehabilitation interventions** |  |  |  |  |
| Case pair 4 | 1.61 (0.58) | 2.22 (0.67) | 7.04 (1.02) | 7.35 (0.94) |
| Case pair 5 | 1.04 (0.71) | 1.43 (0.79) | 7.13 (1.14) | 7.22 (1.17) |
| Case pair 6 | 2.09 (0.67) | 1.78 (0.6) | 7.39 (0.99) | 7.61 (0.89) |
| Average | 1.58 (0.78) | 1.81 (0.75) | 7.19 (1.05) | 7.39 (1.00) |
| Average of all cases | 1.29 (0.89) | 1.69 (0.74) | 7.29 (1.03) | 7.52 (1.01) |

Table 6: LMM models

|  | **Model 1: Cases on determining important RTW barriers** | | **Model 2: Cases on determining suitable RTW interventions** | |
| --- | --- | --- | --- | --- |
|  | B (95% CI) | p-value | B (95% CI) | p-value |
| **Agreement with gold standard** | 0.57 (0.33-0.80) | <0.001 | 0.23 (0.01-0.45) | 0.039 |
| **Confidence in assessment** | 0.26 (-0.00-0.05) | 0.052 | 0.20 (-0.01-0.42) | 0.061 |
